# Understanding the use of artificial intelligence for implant analysis in total joint arthroplasty: a systematic review

**DOI:** 10.1186/s42836-023-00209-z

**Published:** 2023-11-03

**Authors:** Aakash K. Shah, Monish S. Lavu, Christian J. Hecht, Robert J. Burkhart, Atul F. Kamath

**Affiliations:** 1 Department of Orthopaedic Surgery, Cleveland Clinic Foundation, Cleveland, OH 44195 USA; 2 Department of Orthopaedic Surgery, University Hospitals, Cleveland, OH 44106 USA; 3 Center for Hip Preservation, Orthopaedic and Rheumatologic Institute, Cleveland Clinic Foundation, 9500 Euclid Avenue, Mail Code A41, Cleveland, OH 44195 USA

**Keywords:** Artificial intelligence, Total joint arthroplasty, Implant recognition, Implant failure, Measurements

## Abstract

**Introduction:**

In recent years, there has been a significant increase in the development of artificial intelligence (AI) algorithms aimed at reviewing radiographs after total joint arthroplasty (TJA). This disruptive technology is particularly promising in the context of preoperative planning for revision TJA. Yet, the efficacy of AI algorithms regarding TJA implant analysis has not been examined comprehensively.

**Methods:**

PubMed, EBSCO, and Google Scholar electronic databases were utilized to identify all studies evaluating AI algorithms related to TJA implant analysis between 1 January 2000, and 27 February 2023 (PROSPERO study protocol registration: CRD42023403497). The mean methodological index for non-randomized studies score was 20.4 ± 0.6. We reported the accuracy, sensitivity, specificity, positive predictive value, and area under the curve (AUC) for the performance of each outcome measure.

**Results:**

Our initial search yielded 374 articles, and a total of 20 studies with three main use cases were included. Sixteen studies analyzed implant identification, two addressed implant failure, and two addressed implant measurements. Each use case had a median AUC and accuracy above 0.90 and 90%, respectively, indicative of a well-performing AI algorithm. Most studies failed to include explainability methods and conduct external validity testing.

**Conclusion:**

These findings highlight the promising role of AI in recognizing implants in TJA. Preliminary studies have shown strong performance in implant identification, implant failure, and accurately measuring implant dimensions. Future research should follow a standardized guideline to develop and train models and place a strong emphasis on transparency and clarity in reporting results.

**Level of Evidence:**

Level III.

**Supplementary Information:**

The online version contains supplementary material available at 10.1186/s42836-023-00209-z.

## Background

Total hip arthroplasty (THA) and total knee arthroplasty (TKA) are increasingly high-volume orthopaedic procedures expected to grow by 71% and 85% by 2030, respectively [1–4]. This growing population of arthroplasty patients is paired with an increasing volume of total joint arthroplasty (TJA) reoperations [5–8]. Radiographic assessment is the most prevalent method to identify the correct positioning of the implant, monitor implant wear, exclude complications, and identify implant design before revision surgery [9, 10]. However, these analyses place a significant burden upon arthroplasty surgeons. For instance, inconsistent implant records can complicate implant identification before revision surgery, increasing perioperative morbidity and cost of care [11]. In a 2012 survey of arthroplasty surgeons, 88% of respondents claimed that identifying components of a failed implant takes a significant amount of time [12].

Artificial intelligence (AI) presents an alternative to this time-consuming process, and the reduction of human error could further optimize preoperative planning. AI algorithms can extract rules and patterns from large amounts of data to predict outcomes with sets of similar data [13]. Machine learning (ML) and deep learning models, known as convolutional neural networks (CNNs), are subsets of AI modeled after the human brain to identify rules and patterns in images [14–17]. AI algorithms have been utilized to detect mammographic lesions [18], skin cancer [19], and have a growing presence in orthopaedic surgery [14, 15, 17]. AI has been promising in preoperative planning for revision TJA where multiple aspects of the implant need to be analyzed [20, 21].

As the rate of revision TJAs is rising for a multitude of reasons, AI implant recognition may reduce surgeon workload, save resources, and reduce inaccuracies necessitating another revision. Because of the plethora of different AI algorithms, a systematic review of current studies exploring the nature of these algorithms is critical to understanding the efficacy and potential use cases. Therefore, we asked: (1) What are the currently established use cases for AI in TJA? (2) What is the performance of these algorithms? (3) What are the current limitations of these AI algorithms?

## Methods

This review was conducted according to the Preferred Reporting Items for Systematic Reviews (PROSPERO registration of the study protocol: CRD42023403497, 27 February 2023).

### Search strategy

The PubMed, EBSCOhost, Medline, and Google Scholar electronic databases were searched on 27 February 2023, to identify all studies published between 1 January 2000, and 27 February 2023 evaluating AI-mediated implant analysis in hip and knee arthroplasty. The following keywords and Medical Subject Headings were used in combination with the “AND” or “OR” Boolean operators: (“Total Joint Arthroplasty [Mesh]” OR “Total Knee Arthroplasty [Mesh]” OR “Total Hip Arthroplasty [Mesh]” OR “THA” OR “TKA” OR “TJA”) AND (“Artificial Intelligence” OR “AI” OR “Machine Learning” OR “ML”) AND (“Implant”).

### Eligibility criteria

Articles were included if (1) full-text manuscripts in English were available and (2) the study investigated the use of artificial intelligence algorithms in TJA implant analysis. Additionally, the following studies were excluded from our analysis: (1) case reports, (2) systematic reviews, (3) duplicate studies among databases, (4) gray literature such as abstracts and articles on pre-print servers, and (5) publications in languages other than English.

### Study selection

**Fig. 1:**
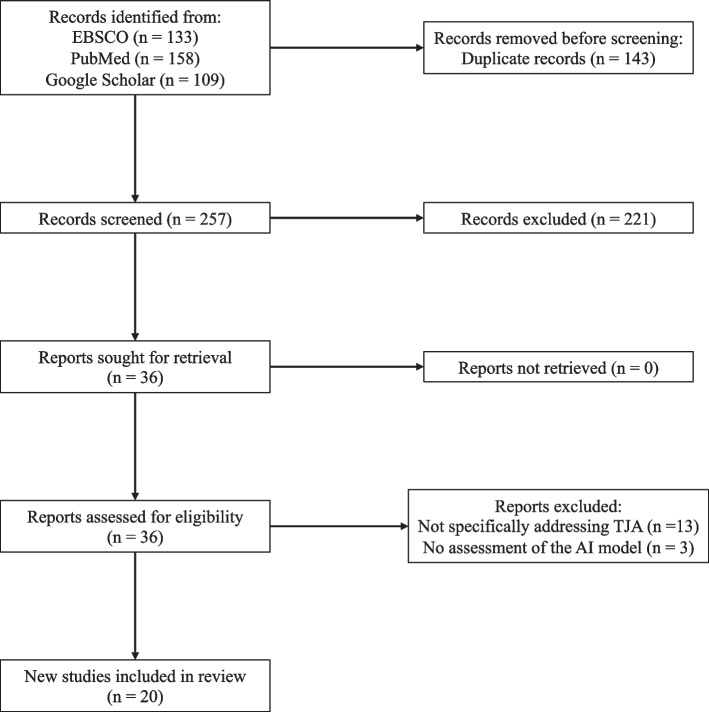
PRISMA diagram depicting the study selection process

Two independent reviewers assessed the eligibility of each included article. Disagreements were discussed with a third independent reviewer to achieve consensus. Upon removing duplicates, the initial query yielded 257 articles, which were then screened for appropriate studies aligning with the purpose of this review. 36 studies were selected for further consideration after the title and abstract screening. The full text of each article was reviewed, 20 of which fulfilled our inclusion criteria. Reasons for full-text exclusion included the study not directly addressing implant analysis in TJA (*n* = 13), and the study not assessing the efficacy of an AI model (*n* = 3). A review of each study’s reference list yielded no additional articles (Fig. 1).

### Study characteristics

A total of 20 studies evaluating 66,190 radiographs were included in the final analysis (Table 1). The efficacy of AI-mediated implant recognition was reported for TKA in 10 studies, for THA in 8 studies, and for both in 2. The included studies were conducted between 2020 and 2023, with all 20 reviewing radiographs retrospectively. While 13 studies were conducted with data from single.

**Table 1:** Characteristics of studies included in the final analysis

**Author**	**Journal**	**Sample Size**	**Number of Implant Designs**	**Image Source**	**Imaging**	**Implant location**	**AI device**	**Purpose**	**Training:** **Validation:** **Testing Split**	**Minors Score**
Belete et al., 2021 [22]	*Informatics in Medicine Unlocked*	558	7	Single Institution	AP Radiograph	Knee	CNN (ResNet)	Implant Identification	50:25:25	20
Bonnin et al., 2023 [23]	*Journal of Arthroplasty*	38,751	4	Single Institution	AP & Lateral Radiograph	Knee	X-TKA, 12 DCNN	Implant Identification	60:20:20	21
Borjali et al., 2020 [24]	*Journal of Orthopaedic Research*	252	3	Single Institution	AP Radiograph	Hip	DCNN	Implant Identification	80:10:10	20
Borjali et al., 2021 [25]	*Medical Physics*	402	9	Single Institution	AP Radiograph	Hip	DCNN	Implant Identification	80:10:10	21
Lau et al., 2022 [37]	*Journal of Orthopaedic Translation*	440	NR	Single Institution	AP Radiograph	Knee	Xception Model	Failure (Loosening)	75:25^a^	21
Ghose et al., 2020 [26]	*ICISS*	878	6	Multi-Center & Textbooks	AP & Lateral Radiograph	Knee	DCNN (various)	Implant Identification	80:10:10	19
Gong et al., 2022 [27]	*Scientific Reports*	714	4	Single Institution	AP Radiograph	Hip (Stem and Cup)	CNN	Implant Identification	60:30:10	20
Jang et al., 2023 [28]	*Journal of Arthroplasty*	235	NR	Single Institution	AP Radiograph	Knee	U-Net Model	Fixation Zone & Cone Mapping Identification	60:20:20	20
Kang et al., 2020 [29]	*Journal of Orthopaedic Translation*	170	29	Multi-Center	AP Radiograph	Hip and Knee	YOLOv3 Object Detection Keras Deep Learning Platform	Implant Identification	75:25^a^	20
Karnuta et al., 2021 [8]	*Journal of Arthroplasty*	682	9	Multi-Center	AP Radiograph	Knee	DCNN (Inception V3)	Implant Identification	80:10:10	21
Karnuta et al., 2021 [7]	*Journal of Arthroplasty*	1972	18	Multi-Center	AP Radiograph	Hip	CNN (InceptionV3)	Implant Identification	80:10:10	20
Karnuta et al., 2022 [21]	*Journal of Arthroplasty*	2954	8	Multi-Center	AP Radiograph	Hip	CNN	Implant Identification	70:10:20	21
Klemt et al., 2022 [30]	*JAAOS*	11,204	24 THA, 14 TKA	Single Institution	AP Radiograph	Hip and Knee	CNN	Implant Identification	80:20^a^	20
Murphy et al., 2022 [36]	*HIP International*	2,440	8	Single Institution	AP Radiograph	Hip	DCNN	Implant Identification	60:30:10	20
Patel et al., 2021 [31]	*Radiology: Artificial Intelligence*	922 THA, 427 TKA	8 THA, 4 TKA	Single Institution	AP Radiograph	Hip and Knee	DCNN (various)	Implant Identification	70:20:10	21
Rouzrokh et al., 2022 [32]	*Radiology Artificial Intelligence*	700	2	Single Institution	AP Radiograph	Hip	U-Net Model	Failure (Subsidence)	70:15:15	21
Schwarz et al., 2022 [33]	*KSSTA*	1,512	8	Single Institution	Long Leg Radiograph	Knee	IB Lab LAMA	Measurements	> 15,000:200:1,312	21
Sharma et al., 2021 [34]	*Indian Journal of Orthopaedics*	1,078	6	Multi-Center	AP & Lateral Radiograph	Knee	DCNN (various)	Implant Identification	75:15:10	20
Tiwari et al., 2022 [20]	*Journal of Orthopaedics*	521	6	Single Institution & Google Images	AP & Lateral Radiograph	Knee	Transfer Machine Learning	Implant Identification	70:20:10	21
Yi et al., 2020 [35]	*The Knee Journal*	274	2	Single Institution & Two Public Datasets	AP Radiograph	Knee	CNN	Implant Identification	70:10:20	20

*CNN* Convolutional Neural Network, *DCNN* Deep Convolutional Neural Network, *ResNet* Residual Network, *AP* Anterior Posterior

^a^No Validation

Institutions, 7 studies utilized data from multiple institutions. All studies were diagnostic trials exploring the efficacy of AI algorithms regarding TJA.

### Risk of bias in individual studies

Two independent reviewers assessed the risk of bias by using the Methodological Index for Nonrandomized Studies (MINORS) tool. This is a validated assessment tool that grades comparative studies from 0 to 24 based on 12 criteria related to study design, outcomes assessed, and follow-up, with higher scores reflecting better study quality. Per domain, each item was scored 0 if low, 1 if moderate, and 2 if high (Supplemental Fig. 1). Discrepancies in grading were resolved by achieving consensus through consulting a third reviewer. The mean MINORS score was 20.4 ± 0.6.

### Primary and secondary outcomes

Firstly, we identified the currently established use cases for AI in TJA implant analysis. These were found to be implanted style identification, implant failure identification, and implant measurement. The primary goal of this study was to present the efficacy of current AI algorithms in implant recognition following total joint arthroplasty. To achieve this, we performed an analysis of the accuracy, the area under the curve (AUC) for the receiver operating characteristic (ROC) curve, sensitivity, specificity, and positive predictive value (PPV) for each use case. The median and interquartile ranges (IQR) were calculated using Excel (Microsoft Corporation, Redmond, Washington, USA) for the highest-scoring AI algorithm in each study. As a secondary goal, we synthesized key limitations that the authors of each study had noted.

## Results

### Implant identification

Most studies (*n* = 16) included in this review explored the efficacy of AI algorithms in identifying implant shape, model, and manufacturer. Seven of these studies were TKA implant-specific, seven were THA implant-specific, and two included implants for both surgeries. For TKA algorithms, the AUC ranged from 0.9857 to 1, accuracy ranged from 22.2% to 100%, sensitivity ranged from 22.2% to 100%, PPV ranged from 22.2% to 100%, and specificity ranged from 97.8% to 100% (Table 2). The median (IQR) for each of these domains was AUC: 0.996 (0.990 to 1), accuracy: 98.9% (96.9% to 99.8%), sensitivity: 98.1% (94.8% to 99.7%), PPV: 99.6% (99.0% to 100%), and specificity: 99.4% (98.1% to 100%). Of note, one study was able to develop an algorithm with perfect scores across all reported domains [35]. For THA algorithms, the AUC ranged from 0.99 to 0.999, accuracy ranged from 83.7% to 100%, sensitivity ranged from 75.4% to 98.90%, PPV ranged from 83.7% to 99.0%, and specificity ranged from 98.0% to 99.80% (Table 3). The median (IQR) for each of these domains was AUC: 0.999 (0.995 to 0.999), accuracy: 98.2% (91.7% to 99.6%), sensitivity: 94.6% (94.3% to 95.7%), PPV: 96.3% (93.1% to 99.0%), and specificity: 99.2% (98.5% to 99.8%).

**Table 2:** Performance of artificial intelligence algorithms in identifying implants for total knee arthroplasty

**Author**	**AI Technique**	**DCNN**	**AUC**	**Accuracy**	**Sensitivity/Recall**	**Precision/PPV**	**Specificity**
Belete et al., 2021 [22]	Hyperparameter, Manual Segmentation Pre-Processing, Data Augmentation	ResNet-18	1	100%	NR	NR	NR
Bonnin et al., 2023 [23]	Exam Quality Control CNN Deep Learning	X-TKA	NR	99.9%	99.8%	100%	100%
Ghose et al. 2020 [26]	Histogram Equalization Data Augmentation, Albumentations Deep Learning DCNN	MobileNetV2	NR	96.7%	NR	NR	NR
Karnuta et al., 2021 [7, 8]	DCNN	InceptionV3	0.992	98.9%	94.6%	94.6%	99.4%
Klemt et al., 2022 [30]	CNN Preprocessing Hyp,erparameter Optimization, Class Activation Heat Maps	InceptionV3	NR	Primary TKA: 97.4% Revision TKA: 96.3%	Primary TKA: 94.9% Revision TKA: 94.5%	NR	Primary TKA: 97.8% Revision TKA: 98.1%
Patel et al., 2021 [31]	DCNN, Hyperparameter Optimization, Image Segmentation/Data Augmentation Ensembled Networks	EfficientNet & U-Net	NR	98.9% Human: 76.1%	98.9%	99%	NR
Sharma et al., 2021 [34]	BRISQUE Data Augmentation Fine-Tuning in Transfer Learning DCNN	ResNet-50v2, VGG16, MobileNetV2, DenseNet-201	0.9857	96.4%	97.20%	NR	NR
Tiwari et al., 2022 [20]	Transfer Machine Learning Models	ResNet-50, MobileNet, Efficient Net B7, InceptionV3, Nasnet, VGG16, Xception, Human	NR	ResNet-50-51.4% MobileNet -99.6% Efficient Net B7 -22.2% InceptionV3-96.2% Nasnet-94.6% VGG16-99.0% Xception-93.1% Human-78.2%	ResNet-50-42.0% MobileNet-99.6% Efficient Net B7-22.2% InceptionV3-96.2% Nasnet-94.6% VGG16-99.0% Xception-93.1% Human-50.0%	ResNet-50-62.0% MobileNet-99.6% Efficient Net B7-22.2% InceptionV3-96.2% Nasnet-94.6% VGG16-99.0% Xception-93.4% Human-80.1%	NR
Yi et al., 2020 [35]	Data Augmentation DCNN	ResNet-18	1	100%	100%	100%	100%
Median (IQR)	NA	NA	0.996 (0.990–1)	98.9% (96.9%–99.8%)	98.1% (94.8%–99.7%)	99.6% (99.0%–100%)	99.4% (98.1%–100%)

*CNN* Convolutional Neural Network, *DCNN* Deep Convolutional Neural Network, *AUC* area under the receiver operating characteristic curve, *PPV* positive predictive power, *ResNet* Residual Network, *SD* standard deviation, *NR* not reported, *NA* not applicable

**Table 3:** Performance of artificial intelligence algorithms in identifying implants for total hip arthroplasty

**Author**	**AI Technique**	**DCNN**	**AUC**	**Accuracy**	**Sensitivity/Recall**	**Precision/PPV**	**Specificity**	**Processing Speed per Radiograph**
Borjali et al., 2020 [24]	DCNN	DenseNet-201	NR	100%	NR	NR	NR	NR
Borjali et al., 2021 [25]	DCNN	DenseNet-201	NR	78% Human: 85%	NR	NR	NR	NR
Gong et al., 2022 [27]	CNN Transfer Learning Framework Backward-Propagation Hyperparameter Tuning Data Augmentation	ResNet-50	NR	Stem network: 91.5% Cup network: 83.7% Combined: 88.6% Joint network: 88.8%	Stem Network: 84.7% Cup Network: 75.4% Combined: 76.5% Joint Network: 82.1%	Stem Network: 91.5% Cup Network: 83.7% Combined: 88.6% Joint Network: 88.8%	NR	NR
Kang et al., 2020 [29]	Image Augmentations Histogram Equalization Flipping Rotating	Keras API	0.99	NR	NR	> 99%	NR	NR
Karnuta et al., 2021 [7, 8]	CNN Class Activation Heatmap	InceptionV3	0.999	99.60%	94.3%	NR	99.8%	NR
Karnuta et al., 2022 [21]	Image Preprocessing CNN Development	CNN	0.999	99.6	94.3%	93.6%	99.8%	0.02 s
Klemt et al., 2022 [30]	CNN Preprocessing Hyperparameter Optimization, Class Activation Heat Maps	InceptionV3	NR	Primary THA: 98.2% Revision THA: 98.0%	Primary THA: 95.8% Revision THA: 94.9%	NR	Primary THA: 98.6% Revision THA: 98.0%	NR
Murphy et al., 2022 [36]	Dropout and Batch Normalization Techniques	DenseNet-201	NR	91.7%	NR	NR	NR	0.96 ± 0.02 s
Patel et al., 2021 [31]	DCNN, Hyperparameter Optimization, Image Segmentation/Data Augmentation Ensembled Networks	EfficientNet & U-Net	NR	98.9% Human: 76.1%	98.90%	99%	NR	0.06 s vs Surgeon: 8.4 ± 6.1 min
Median (IQR)	NA	NA	0.999 (0.995–0.999)	98.2% (91.7%–99.6%)	94.6% (94.3%–95.7%)	96.3% (93.1%–99.0%)	99.2% (98.5%–99.8%)	0.06 (0.04–0.51)

*CNN* Convolutional Neural Network, *DCNN* Deep Convolutional Neural Network, *AUC* area under the receiver operating characteristic curve, *PPV* positive predictive power, *ResNet* Residual Network, *SD* standard deviation, *NR* not reported, *NA* not applicable, *s* seconds, *min* minutes

Additionally, three studies were able to compare the identification capabilities of AI relative to that of a human expert [20, 25, 31]. Of these three studies, two showed improved performance from a certain AI architecture when compared to arthroplasty clinicians [20, 31]. However, one study showed poorer performance from their AI architecture when compared to experts [25]. Three studies also reported the average time spent per radiograph by their algorithm, which was less than one second [21, 25, 36]. In comparison, one study reported the time required for a surgeon to analyze a radiograph with which they had no experience to be greater than eight minutes [36].

The most common limitation noted by authors was the limited dataset upon which the algorithms were trained [7, 8, 22, 24–27, 29, 35]. In addition to a limited number of radiographs, authors also faced challenges with developing an algorithm with generalizability due to a limited library of implants [7, 8, 20–22, 26, 27, 30, 31, 35, 36]. authors noted a lack of high-quality radiographs of implants from various imaging positions and modalities [7, 29, 30, 35], which further hampers their generalizability. Lastly, authors advocated for a need to validate these algorithms through comparison with the judgment of both surgeons of varying experience [23, 27, 30].

### Implant failure detection

Two studies aimed to detect implant failure through the utilization of AI algorithms [32, 37] (Table 4). One study sought to assess implant loosening in TKA [37]. When compared to the baselines set by two orthopaedic specialists, the image-based algorithm attained an accuracy of 96.3% with no improvement upon adding clinical information. Additionally, class activation maps (CAMs) showed signals over the loosened bone-implant interface**,** the parameters for detecting implant loosening. The other study developed a deep learning tool to quantify femoral component subsidence between serial AP radiographs of the hip [32]. Parameters included distance from the tip of the stem to the most superior point on the greater trochanter, angle of the femoral axis, and distance between magnification markers. The model was able to achieve an accuracy of 97% for detecting the femur, 98% for detecting the implant, and 94% for detecting the magnification markers. When compared to the manual measurements of two orthopaedic surgeon reviewers, the automatic measurements had an absolute mean error of 0.6 (21%) ± 0.7 mm. The measurements bore a strong correlation of 0.96 (*P* < 0.001). The median (IQR, if applicable) for implant failure detection algorithms was AUC: 0.935, accuracy: 97.2% (96.7%–97.6%), sensitivity: 96.1%, PPV: 92.4%, and specificity: 90.9%.

**Table 4:** Performance of artificial intelligence algorithms detecting implant failure in total joint arthroplasty

**Author**	**AI Technique**	**DCNN**	**AUC**	**Accuracy**	**Sensitivity/Recall**	**Precision/PPV**	**Specificity**
Lau et al., 2022 [37]	Pre-Trained on ImageNet and Tensor Flow	Xception Model	0.935	96.3%	96.1%	92.4%	90.9%
Rouzrokh et al., 2022 [32]	U-Net Model	Efficient Net B0	NR	Femur-97.0% Implant-98.0% Magnification Markers: 94.0%	NR	NR	NR
Median (IQR)	NA	NA	0.935 (NA)	97.2% (96.7%–97.6%)	96.1% (NA)	92.4% (NA)	90.9% (NA)

*DCNN* Deep Convolutional Neural Network, *AUC* area under the receiver operating characteristic curve, *PPV* positive predictive power, *SD* standard deviation, *NR* not reported, *NA* not applicable

Both studies acknowledged similar limitations: small datasets, the use of cemented implants limiting external validity as the use of cementless implants is rising, and alterations in the radiographic appearance of bones due to heterotopic ossification, bisphosphonate administration, and magnesium coatings over implants [32, 37].

### Implant measurement

Two studies assessed the measurement capabilities of AI in total joint arthroplasty [28, 33] (Table 5). In one study, the authors attempted to build an algorithm to delineate the epiphyseal, metaphyseal, and diaphyseal fixation zones and cone placements following revision TKA [28]. To accomplish this, the widest condylar width, most inferior points of the femoral implant, widest tibial width, and most proximal points of the tibial implant were used as parameters to construct squares on the femur and tibia. 98% of zones were able to be delineated, and when compared to a fellowship-trained orthopaedic surgeon, the algorithm achieved a 90% zonal mapping accuracy, with 97.8% tibial and 100% femoral cone identification. Runtime for the algorithm was 8 ± 0.3 s per radiograph [28]. In another study, an algorithm was trained on long leg radiographs (LLR) following TKA to assess the alignment of knee systems with reads of the hip-knee-ankle (HKA), femur component (FCA), and tibial component (TCA) angles [33]. This study was conducted using the commercially available AI software IB Lab LAMA (Leg Angle Measurement Assistant, version 1.03, IB Lab GmbH, Vienna, Austria), which localizes anatomical features of the femur, tibia, and calibration ball to measure leg angles. When compared to two orthopaedic surgeons who regularly perform LLR measurements, the algorithm achieved an accuracy of 99% for HKA, 99% for FCA, and 97% for TCA. For these measurement studies, the median (standard deviation) of the highest accuracy achieved was 97.3% (94.5% to 99.3%). Noted limitations included limited knee systems for algorithm training and limited cohorts for external validation, especially those with varying degrees of image quality [28, 33].

**Table 5:** Performance of artificial intelligence algorithms measuring implants in total joint arthroplasty

**Author**	**AI Technique**	**DCNN**	**AUC**	**Accuracy**	**Sensitivity/Recall**	**Precision/PPV**	**Specificity**
Jang et al., 2023 [28]	CNN Transfer Learning to Segment Relevant Landmarks	U-Net Model	NR	Zonal Mapping: Femoral-89% Tibial-91% All Zones-90% Cone Identification: Femoral-97.8% Tibial-100% Cone Placement: Femoral-95.7% Tibial-89.1%	NR	NR	NR
Schwarz et al., 2022 [33]	IB Lab LAMA	NR	NR	HKA: 99% FCA: 99% TCA: 97%	NR	NR	NR
Median (IQR)	NA	NA	NA	97.3% (94.5%–99.3%)	NA	NA	NA

*CNN* Convolutional Neural Network, *DCNN* Deep Convolutional Neural Network, *AUC* area under the receiver operating characteristic curve, *PPV* positive predictive power, *SD* standard deviation, *NR* not reported, *NA* not applicable, *HKA* hip-knee-ankle angle, *FCA* femoral component angle, *TCA* tibial component angle

## Discussion

AI algorithms for TJA implant analysis have shown promising preliminary results regarding identification, failure detection, and measurement. For all these use cases, algorithms have been able to demonstrate high accuracy, PPV, sensitivity, and specificity. Some studies were also able to demonstrate that these algorithms could outperform human experts. Yet still, a major limitation noted by almost all studies was a limited radiographic dataset size which limits their extrapolation, as the AI needs to be trained on all types of inputs it is expected to perform upon. Overall, AI algorithms show promise in implant identification, failure detection, and measurement with the ability to improve orthopaedic workflow similar to prior integrations of AI into workflows [38–41]. For wider implementation and validation of AI, future algorithms need to be trained on a robust set of high-quality datasets, externally validated, and publish explainability methods.

The lack of robust and high-quality datasets has been identified as a significant limitation in multiple studies, adversely affecting the performance of AI algorithms. Consequently, some of these studies failed to meet the desired thresholds for excellent algorithm performance, namely an AUC of 0.90 and an accuracy of 90%. The performance of algorithms that did not have access to a large dataset of high-quality images will most likely worsen when externally validated [42–44]. Nonetheless, the approximate volume of imaging samples needed for high sensitivity and specificity can be relatively low (< 500). All but one of the studies reporting these metrics [27] were able to achieve high sensitivity and specificity for implant identification even though the image sample sizes ranged from 274 to 11,204 images in total. Even when considering implant design, a very low quantity of images per design is required. Many studies used augmentation techniques to increase the number of images for training through contrast editing, flipping, and rotating of raw image data. Through this technique, Kang et al. were able to create 3606 augmented images from 179 images of 29 hip implant designs with some having less than 5 radiographs and still achieve an AUC of 0.99 [29]. However, even algorithms that demonstrated excellent performance are limited by the catalog of implants and radiographs presented to them. To improve the AUC and accuracy of future studies, high-quantity and high-quality datasets need to be publicly available [45]. Datasets including all training images from DICOM to standard JPG formats would be beneficial to allow for AI training on multiple image mediums. Few well-curated imaging datasets are currently available due to a lack of image organization, anonymization, annotation, and linkage to a ground-truth diagnosis [45].

Institutional-level datasets limit the ability for external validation. As a result, fourteen out of the twenty included studies did not test their algorithms against an external dataset, making it difficult to understand if these results are reproducible in a different environment [46]. Park and Han stress the importance of testing all algorithms against a well-defined clinical cohort to eliminate potential overestimation of the algorithms' performance due to overfitting or overparameterization [47]. Failing to test against external datasets is not uncommon, with only 6% of prior radiological AI papers using an external test set [48]. To improve the reproducibility of the AI algorithms, future studies ought to conduct tests against external datasets [38, 42, 43, 47, 49, 50]. Developers ought to consider the external validity of the algorithm and minimize the risk of overestimation by testing with an external dataset and utilizing a strongly defined clinical cohort, respectively. Developers will have greater success at the institutional level compared to the global scale due to the vast library of implants that joint reconstructive surgeons use. A long-term solution for these concerns would be to create an implant library that any reconstructive surgeon at any institution could utilize to create new algorithms. While external validity is a concern for these algorithms, the internal validity is still very high so developers can create institution-specific algorithms based on the catalog of implants that their reconstructive surgeon routinely uses. With algorithmic training on high-quality publicly available datasets and external testing, the clinical feasibility of these algorithms may be better assessed.

Lastly, AI models have a “black box” phenomenon as most users are unable to understand how the algorithm reaches its decision. This phenomenon has been faced with criticism on whether or not to trust AI as one cannot trace the logic [51]. Saliency mapping and CAMs are methods to explain the region of the image that was relevant in the algorithm’s decision [52]. For example, a saliency map for identifying THA implants disclosed that the region around the tip of the femoral component was of utmost importance, something which has not been commonly used as a distinguishing factor between models [24]. However, these maps may not be enough as a few studies included in this review [22, 24, 26, 31, 34] demonstrated that AI-based implant measurement and failure detection require various other parameters. Therefore, all future studies should report the parameters as well as the saliency maps associated with decision-making to improve the transparency of the AI algorithms for potential clinician adopters.

### Limitations

This study has its limitations. Firstly, not all values for AUC, accuracy, sensitivity, PPV, and specificity were included. The variation in performance reporting limits the accuracy of generalizations regarding the performance of these algorithms. Along these lines, the algorithms each have their own library of implants upon which they were trained. Due to this, overarching comparisons between studies are difficult to make as the algorithms were tested upon different images and implants. Additionally, very few studies reported demographic information corresponding to radiographic datasets. This will be crucial in the future as biased clinical data will negatively affect model performance [53]. Nonetheless, the results reported in included studies show promising results for AI-based implant analysis.

## Conclusion

AI models hold great potential as a disruptive tool in the field of adult reconstructive surgery, specifically in the analysis of implants. This is particularly important considering the rising demand for revision TJA. AI-based implant analysis can reduce the workload of surgeons, save resources, and minimize inaccuracies that might necessitate further revisions. These findings highlight the promising role of AI in recognizing implants in TJA. Initial studies have demonstrated impressive performance in implant classification, analysis of implant failures, and measurements derived from radiographs. However, to develop more robust models, it is essential to have access to larger datasets of radiographs. Future research should adhere to standardized guidelines for model development and training while emphasizing the importance of transparency in presenting the results.

### Supplementary Information

**Additional file 1.**

## Data Availability

Not applicable.
